# Nasal vaccination of triple-RBD scaffold protein with flagellin elicits long-term protection against SARS-CoV-2 variants including JN.1

**DOI:** 10.1038/s41392-024-01822-3

**Published:** 2024-04-27

**Authors:** Xian Li, Mengxin Xu, Jingyi Yang, Li Zhou, Lin Liu, Min Li, Shasha Wang, Mei-Qin Liu, Zhixiang Huang, Zhen Zhang, Shuning Liu, Yunqi Hu, Haofeng Lin, Bowen Liu, Ying Sun, Qingguo Wu, Zheng-Li Shi, Ke Lan, Yu Chen, Huimin Yan, Yao-Qing Chen

**Affiliations:** 1 Wuhan Institute of Virology, Chinese Academy of Sciences, Wuhan, China; 2 Vaccine and Immunology Research Center, Translational Medical Research Institute, Shanghai Public Health Clinical Center, Fudan University, Shanghai, China; 3 University of Chinese Academy of Sciences, Beijing, China; 4 School of Public Health (Shenzhen), Shenzhen Campus of Sun Yat-sen University, Shenzhen, Guangdong China; 5 State Key Laboratory of Virology, Modern Virology Research Center, College of Life Sciences, Wuhan University, Wuhan, China; 6 Aerosol Bio-Tech (Suzhou) Co., LTD, Suzhou, Jiangsu China; 7 National Medical Products Administration Key Laboratory for Quality Monitoring and Evaluation of Vaccines and Biological Products, Sun Yat-sen University, Guanzhou, China

**Keywords:** Vaccines, Vaccines, Infectious diseases

## Abstract

Developing a mucosal vaccine against SARS-CoV-2 is critical for combatting the epidemic. Here, we investigated long-term immune responses and protection against SARS-CoV-2 for the intranasal vaccination of a triple receptor-binding domain (RBD) scaffold protein (3R-NC) adjuvanted with a flagellin protein (KFD) (*3R-NC* + *KFDi.n*). In mice, the vaccination elicited RBD-specific broad-neutralizing antibody responses in both serum and mucosal sites sustained at high level over a year. This long-lasting humoral immunity was correlated with the presence of long-lived RBD-specific IgG- and IgA-producing plasma cells, alongside the Th17 and Tfh17-biased T-cell responses driven by the KFD adjuvant. Based upon these preclinical findings, an open labeled clinical trial was conducted in individuals who had been primed with the inactivated SARS-CoV-2 (IAV) vaccine. With a favorable safety profile, the *3R-NC* + *KFDi.n* boost elicited enduring broad-neutralizing IgG in plasma and IgA in salivary secretions. To meet the challenge of frequently emerged variants, we further designed an updated triple-RBD scaffold protein with mutated RBD combinations, which can induce adaptable antibody responses to neutralize the newly emerging variants, including JN.1. Our findings highlight the potential of the KFD-adjuvanted triple-RBD scaffold protein is a promising prototype for the development of a mucosal vaccine against SARS-CoV-2 infection.

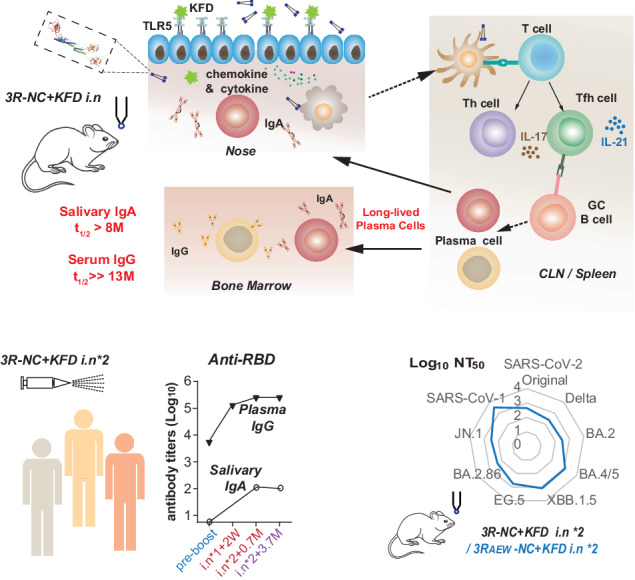

## Introduction

While current SARS-CoV-2 vaccines show promising efficacy in reducing severe illness, their capacity to prevent SARS-CoV-2 infection falls short of expectations.^1,2^ Accumulated mutations make the current circulating JN.1 variant more adaptable at evading the protective immune response elicited by vaccination or previous infection.^3^ There remains an urgent need to develop the next-generation vaccines to counter potential subsequent rounds of SARS-CoV-2 infection and transmission.^4^

The ideal next-generation SARS-CoV-2 vaccines should address three critical challenges simultaneously. Firstly, high level of cross-protection against the rapidly emerging SARS-CoV-2 variants should be generated.^5^ To achieve broad-spectrum protective immunity, multiple approaches have been explored, such as targeting conserved non-neutralizing epitopes,^6^ and employing chimeric or multivalent proteins of receptor-binding domain (RBD), S1, or S.^7,8^ Secondly, protective mucosal immunity should be established in the upper respiratory tract, given that initial infection and replication primarily occur in the nasal ciliated cells.^9^ For this purpose, two inhaled adenovirus-vectored vaccine and one nasal-sprayed influenza-vectored vaccine have received emergency approval in China and Russia.^10,11^ In contrary, no subunit mucosal vaccines against SARS-CoV-2 was approved for clinical use. One critical obstacle in developing subunit mucosal vaccines is the lack of approved mucosal adjuvant for clinical use. Thirdly, protective immunity, particularly the neutralizing antibodies should endure over time. However, this is still a formidable challenge due to the short incubation time of SARS-CoV-2 for recalling adequate memory responses to protect against initial infection.^4^

Some viruses, such as measles virus, can easily induce life-long protective immunity.^12^ However, the neutralizing antibodies against SARS-CoV-2 decline rapidly in humans, no matter elicited by infection or vaccination.^4,13,14^ Even in mouse models, neutralizing antibodies elicited by vaccination could hardly sustain at high level beyond 6 months.^15^ Long-lasting antibody responses are believed associating with the long-lived plasma cells (LLPCs).^16^ Although some factors were found essential for the development and maintenance of LLPCs,^16–18^ and novel markers of LLPC such as EpCAM and Tigit have been identified in recent years,^19^ how to induce large amount of LLPCs by vaccination is still illusive. Hence, repeatedly booster shots were currently recommended to achieve long-term protection against SARS-CoV-2.^20,21^ Another alternative approach to enhance immunity is to use more potent adjuvant.^22^

Adjuvants play a pivotal role not only in eliciting a robust humoral immune response, but also in fostering effective mucosal immune responses. Toll-like receptor 5 (TLR5) agonist, the recombinant flagellin, has been intensively investigated and optimized as a safe and effective mucosal adjuvant in animal model.^23,24^ In developing mucosal vaccines for dental caries,^25^ we observed that the flagellin adjuvanted subunit mucosal vaccines can elicit enduring antibodies, lasting up to 13 months in mice (Supplementary Fig. 1). This observation inspired us to contemplate whether a similar approach could be employed to generate long-effective mucosal vaccines against SARS-CoV-2.

In this study, we employed a highly stable triple-RBD scaffold protein platform capable of accommodating various combinations of three RBDs.^26^ Two novel triple-RBD scaffold proteins, namely 3R-NC and 3R_AEW_-NC, were designed as immunogens for SARS-CoV-2 mucosal vaccines to address the persistent challenge of frequently emerging variants. Nasal vaccination of 3R-NC with an optimized flagellin adjuvant KFD (*3R-NC* + *KFDi.n*), triggered high-level, long-lasting, and broad cross-reactive RBD-specific neutralizing antibodies, in both serum and mucosal sites. These enduring neutralizing antibodies were generated by LLPCs in the bone marrow and nose, which were closely correlated with Th17 and Tfh17 biased immune responses induced by *3R-NC* + *KFDi.n*. Importantly, the triple-RBD scaffold design demonstrated adaptability to new variants, as demonstrated by successful incorporation of the BA.2.86 RBD. Moreover, an investigator-initiated human clinical study demonstrated the promising safety and efficacy of this vaccination strategy. Collectively, our results suggest that the KFD-adjuvanted triple-RBD scaffold subunit mucosal vaccine hold broad-spectrum protection against emerging SARS-CoV-2 variants.

## Results

### Immunogen design and screening of 3R-NC for mucosal immunization

**Fig. 1:**
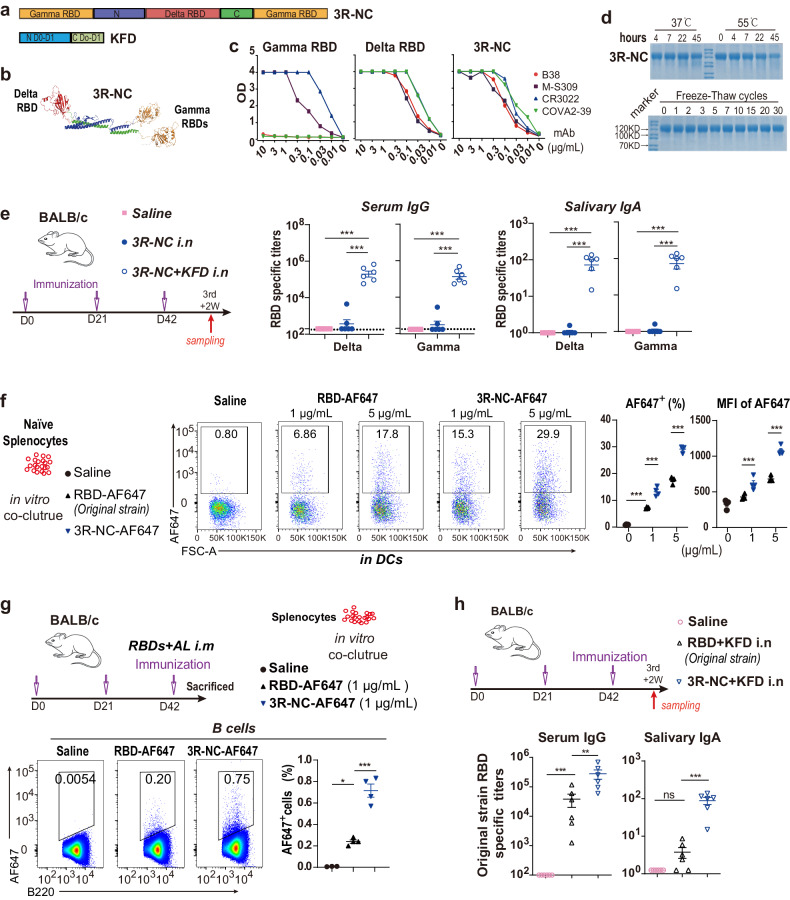
Triple-RBD contained protein (3R-NC) exhibits remarkable stability and high antigenicity potential. **a** Schematic diagram of the flagellin derived adjuvant KFD and chimeric protein 3R-NC. 3R-NC contains one RBD of SARS-CoV-2 Delta strain and two RBDs of Gamma strain, linked together by scaffold NC. **b** 3D structure of the chimeric protein 3R-NC predicted by Alpha Fold 2. **c** ELISA analyzed binding ability of Delta strain RBD, Gamma strain RBD and 3R-NC with four representative classes of neutralizing monoclonal antibodies (mAbs) (*n* = 3). **d** SDS-PAGE analysis of the stability of purified protein 3R-NC in resistance to high temperature and freeze-thaw cycles. **e** RBD-specific antibody responses in BALB/c mice post the 3rd intranasal immunization of 4 µg 3R-NC with or without 1 µg KFD (*3R-NC* + *KFDi.n* vs. *3R-NCi.n*, *n* = 6 mice per group). **f** The uptake ability of the AF-647 labeled RBD or 3R-NC by splenic dendritic cells (DCs). After incubation with naïve BLAB/c mice splenocytes in vitro at 37 °C for 3 h, antigen uptake by DCs were tested by FACS (*n* = 4 per group). **g** The binding ability of AF-647 labeled RBD and 3R-NC by splenic B cells. After 3rd immunization of RBDs with AL adjuvant intramuscularly, splenocytes were separated and incubated with the AF-647 labeled RBD or 3R-NC at 37 °C for 3 h. Then antigen uptake by B cells were tested by FACS (*n* = 4 per group). **h** Antibody responses post the 3rd intranasal immunization of *3R-NC* + *KFDi.n* (4 µg 3R-NC plus 1 µg KFD) and *RBD* + *KFDi.n* (8 µg RBD plus 2 µg KFD) (*n* = 5 mice per group). Data are represented as mean ± SEM and are representative of two independent experiments. Groups were compared using one-way ANOVA. *p < 0.05; ***p < 0.001; ns non-significant

To overcome the limited immunogenicity of RBD antigen and to induce broad-spectrum immune responses, we generated and screened a set of RBD based recombinant proteins on the N-terminal domain and C-terminal domain of scaffold NC (Fig. 1a, b and Supplementary Fig. 2a). Among these candidates, 3R-NC, comprising one Delta strain and two Gamma strain RBDs, possesses unique properties as an antigen. Firstly, 3R-NC likely maintains all four classes neutralizing epitopes of native RBD. This is supported by the high binding affinity of 3R-NC to all the four classes of RBD-specific neutralizing mAbs (Fig. 1c and Supplementary Fig. 2b, c).^27^ Secondly, 3R-NC displayed robust stability against elevated temperatures (37 °C and 55 °C) and maintained its structural integrity even after undergoing repeated freeze-thaw cycles, up to at least 30 cycles (Fig. 1d and Supplementary Fig. 2d).

3R-NC possesses no TLR5-stimulating activity, though it contains flagellin components (NC) as our previous study of 3Ro-NC reported (Supplementary Fig. 2e).^26,28^ Consistent with the defective of the TLR5 agonist, intranasal immunization of 4 µg 3R-NC without mucosal adjuvant could hardly induce RBD-specific serum IgG and salivary IgA (Fig. 1e).

We wondered whether the properties of larger molecular size and triple-RBD containing might be beneficial or detrimental for the antigen uptake and B cell activation. To explore this, Alexa Fluor™647 (AF-647) were labeled onto 3R-NC and RBD monomer separately for antigen tracking. FACscan showed that splenic dendritic cells (DCs) efficiently took up 3R-NC-AF647 in comparison to RBD-AF647, despite 3R-NC-AF647 having only a quarter of the molecular concentration (Fig. 1f and Supplementary Fig. 2f, g). Additionally, in the presence of DCs, the trivalent property of 3R-NC allowed it to bind more efficiently to B cells compared to monomer RBD, as demonstrated by our assays on the splenic cells derived from the RBD immunized mice (Fig. 1g). Consistently, intranasal immunization of 4 µg 3R-NC with 1 µg KFD (*3R-NC* + *KFDi.n*) in mice induced ~10 times higher level of RBD-specific serum IgG and salivary IgA compared to intranasal immunization of 8 µg RBD with 2 µg KFD (*RBD* + *KFDi.n*) (Fig. 1h), despite *3R-NC* + *KFDi.n* contained lower amount of both parts of the target antigen and the adjuvant. Similar to our previous reports,^24,29^ upon uptake by DCs in the nasal region, the antigen-loaded DCs would become activated and migrate to the CLN (Supplementary Fig. 3a). All of these indicated the promising attributes of 3R-NC as antigen for mucosal vaccine.

### Broad protection against SARS-CoV-2 variants induced by *3R-NC* + *KFDi.n*

**Fig. 2:**
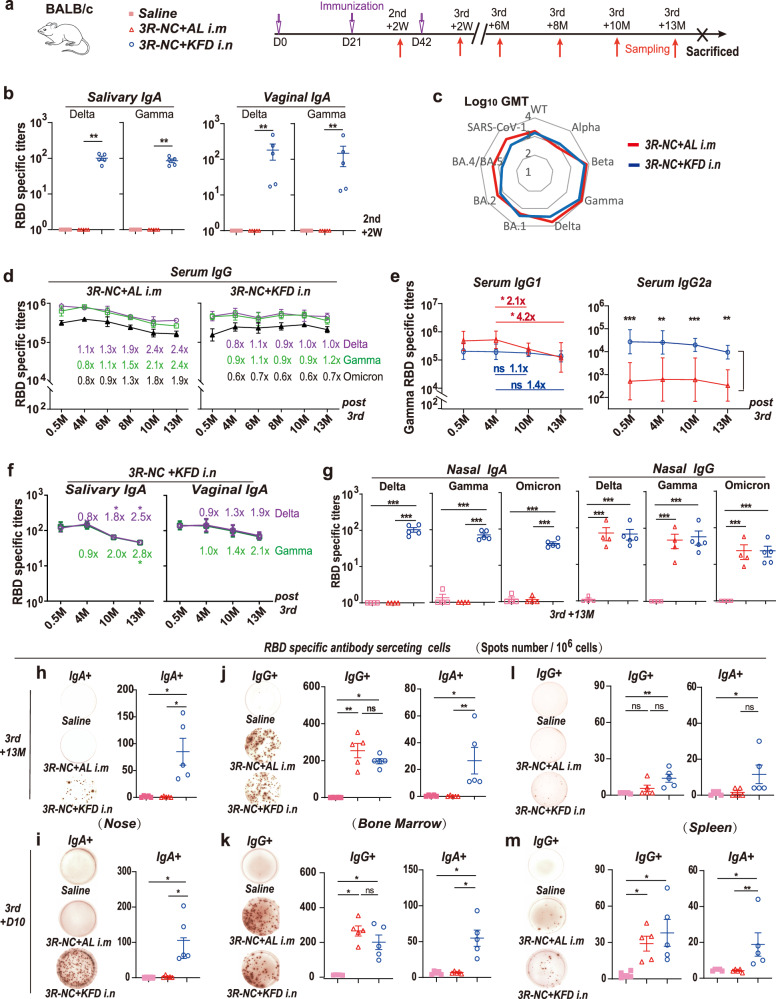
Long-lasting RBD-specific antibodies responses and antibody secreting cells in BALB/c mice. **a** Diagram scheme of immunization and sampling (*n* = 5 mice per group). **b** RBD-specific IgA responses in saliva and vaginal lavage fluid post the 2nd immunization. **c** Geometric mean titers of neutralizing antibody in serum against pseudo-typed SARS-CoV-2 variants or SARS-CoV-1 at day 14 post the 3rd immunization. **d**–**f** RBD-specific serum IgG, serum IgG1, serum IgG2a, salivary IgA and vaginal IgA were measured at various time points post 3rd immunization. **g** RBD-specific IgG and IgA in nasal wash at 13 months post 3rd immunization. ELISPOT assayed RBD-specific IgG and IgA secreting cells at 13 months (**h**, **j**, **l**) and 10 days (**i**, **k**, **m**) post 3rd immunization of *3R-NC* + *KFDi.n* and *3R-NC* + *ALi.m*. Data are represented as mean ± SEM and are representative of two independent experiments. Groups were compared using one-way ANOVA. *p < 0.05; **p < 0.01; ***p < 0.001; ns non-significant

To further assess the characteristics of the immune responses induced by *3R-NC* + *KFDi.n*, intramuscular administration of 3R-NC was selected for comparison. Since intramuscularly administered KFD did not demonstrate robust adjuvanticity (Supplementary Fig. 3b), we opted for the AL adjuvant in subsequent studies. BALB/c mice were immunized by 4 µg 3R-NC with 1 µg KFD intranasally *(3R-NC* + *KFDi.n)* or with 100 µg AL adjuvant intramuscularly (*3R-NC* + *ALi.m*) (Fig. 2a). Notably, only *3R-NC* + *KFDi.n* significantly induced the RBD-specific salivary IgA and virginal IgA responses (Fig. 2b). The sera from both *3R-NC* + *KFDi.n* and *3R-NC* + *ALi.m* both showed broad spectrum to neutralize SARS-CoV-2 variants of concern (VOCs), and even against SARS-CoV-1 (Fig. 2c).

In the human ACE2 transgenic mice (hACE2), *3R-NC* + *KFDi.n* also successfully induced RBD-specific salivary IgA (Supplementary Fig. 4). In addition, *3R-NC* + *KFDi.n* developed even higher RBD-specific IgG and neutralizing antibodies in serum than *3R-NC* + *ALi.m*, offering enhanced and broader protection against challenges from the SARS-CoV-2 Omicron BA.1 strain (Supplementary Fig. 4). All of these findings indicated that *3R-NC* + *KFDi.n* can provide cross-protection against infection of SARS-CoV-2 variant.

### Thirteen-month long-lasting RBD-specific antibodies responses induced by *3R-NC* + *KFDi.n*

We monitored the immune responses up to 13-months post-immunization and observed remarkable enduring RBD-specific IgG responses (Fig. 2d). Specifically, in the *3R-NC* + *KFDi.n* group, the RBD-specific IgG against VOCs exhibited no significant reduction even at 13 months post the 3rd immunization (3rd + 13 M) (Fig. 2d, right panel). In comparison to *3R-NC* + *ALi.m*, *3R-NC* + *KFDi.n* induced comparable levels of RBD-specific IgG1, but significantly higher levels of RBD-specific IgG2a (Fig. 2e). Worth mentioning is the fact that RBD-specific IgG1 induced by *3R-NC* + *KFDi.n* maintained at the same level within 13 months, while that induced by *3R-NC* + *ALi.m* displayed a decreasing trend. Furthermore, RBD-specific IgA responses detected in *3R-NC* + *KFDi.n* group in saliva and virginal lavage fluid were also long-lasting, with only 2 to 3 times reduction within 13 months (Fig. 2f). Even at 3rd + 13 M, the RBD-specific IgA and IgG responses in the nasal lavage fluid (NLF) against SARS-CoV-2 VOCs remained at high levels in *3R-NC* + *KFDi.n* group (Fig. 2g), and retained their neutralizing ability against various variants (Supplementary Fig. 5a). In contrast to the NLF, the titers of RBD-specific IgA responses in the bronchoalveolar lavage fluid (BALF) of the *3R-NC* + *KFDi.n* group were ~93-fold lower than those of RBD-specific IgG (Supplementary Fig. 5b).

In the *3R-NC* + *KFDi.n* group, consistent with the high levels of RBD-specific serum IgG and mucosal IgA, a significant number of RBD-specific IgG secreting cells in bone marrows and RBD-specific IgA secreting cells in the nose tissue were detected at 3rd + 13 M, comparable to the levels observed at 3rd + 10D (Fig. 2h–k). In bone marrows and spleens, RBD-specific IgA secreting cells also long-term existed at 3rd + 13 M, though to a slightly lesser extend (Fig. 2j–m). Nevertheless, IgA-secreting cells in the bone marrows predominantly produced monomer IgA, in contrast to IgA-secreting cells in nasal tissue, which primarily generated dimeric or polymeric IgA (Supplementary Fig. 5c, d). These findings suggested that *3R-NC* + *KFDi.n* can induce a substantial number of long-lasting plasma cells (LLPCs), resulting in the sustained production of RBD-specific antibodies.

### Th17-biased responses associating with the long-lived plasma cells

We further investigated the enduring immune response induced by *3R-NC* + *KFDi.n* immunization. At 3rd + 10D, alongside the presence of 3R-NC specific CD138^+^ B220^−^ plasma cells and CD138^+^ B220^+^ plasma cells in the spleens of *3R-NC* + *KFDi.n* group, 3R-NC specific germinal center (GC) B cells were also detected (Supplementary Fig. 6). These observations suggest that T cells, especially follicle helper T cells (Tfh), might be involved in B cell differentiation.

**Fig. 3:**
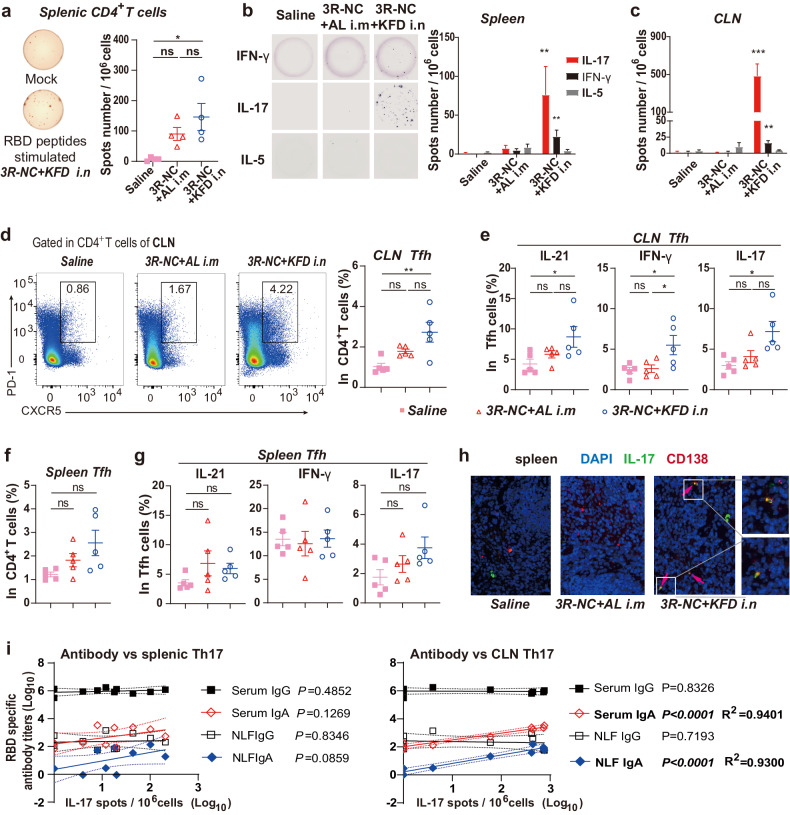
T cells responses in spleen and cervical lymph nodes (CLNs). BALB/c mice were immunized by *3R-NC* + *KFDi.n* and *3R-NC* + *ALi.m* 3 times at 3 weeks interval (*n* = 5 mice per group). T cell responses were tested at day 10 post 3rd immunization. **a** 1.25 × 10^5^ of positive selected CD4^+^ T cells were mixed with 7.5 × 10^5^ naïve splenocyte as antigen presenting cells, and stimulated with or without RBD peptides. RBD-specific IFN-γ secretions were indicated by the mock (unstim) background-normalized spots (*n* = 4 mice per group). **b**, **c** ELISPOT tested the RBD peptides stimulated IFN-γ, IL-17 and IL-5 secretion by cells of spleens and CLNs. **d**, **f** Percentages of Tfh in CD4^+^ T cells of CLNs and spleens. **e**, **g** IL-21, IFN-γ and IL-17 secretion potential of Tfh in CLNs and spleens post non-specific stimulation. **h** Immune-staining of IL-17 (green), plasma cell marker CD138 (red) and cell nuclei (DAPI, blue) in spleens. The co-localization of IL-17 with CD138 were labeled by pink arrow. **i** Correlation of RBD-specific Th17 responses with the RBD-specific IgG and IgA response in mice of *3R-NC* + *KFDi.n* and *3R-NC* + *ALi.m* groups at day 10 post the 3rd immunization. The 95% confidence interval is indicated by dotted lines. Data are represented as mean ± SEM and are representative of two independent experiments. Groups were compared using one-way ANOVA except in (**i**). *p < 0.05; **p < 0.01; ***p < 0.001; ns non-significant

In the spleens of *3R-NC* + *KFDi.n* immunized mice, RBD-specific IFN-γ secretion CD4^+^ T cells responses were readily detected at 3rd + 10D while was considerably lower than that of IL-17A responses (Fig. 3a, b). Similar RBD-specific IFN-γ-secreting T cell responses were observed in the cervical lymph nodes (CLNs) at 3rd + 10D, but Th17-biased responses were even more pronounced in the CLNs than in the spleen (Fig. 3c). In contrast, there were limited Th1 and minimal Th17 responses in the *3R-NC* + *ALi.m* immunized mice (Fig. 3a–c). All these data collectively suggested that *3R-NC* + *KFDi.n* induced a Th17-biased response in the draining lymph nodes and spleen, with a less extend of Th1 response.

Further investigation of Tfh responses revealed a significant up-regulation of Tfh in the CLNs of the *3R-NC* + *KFDi.n* immunized mice (Fig. 3d). Among the CLN Tfh, the potential for secreting IL-21, IFN-γ and IL-17 were also up-regulated in the *3R-NC* + *KFDi.n* immunized mice (Fig. 3e). These indicated *3R-NC* + *KFDi.n* promoted Tfh response in the draining lymph nodes. In the spleens, up-regulation of Tfh and the secreting potential of IL-21 and IL-17 in Tfh in the *3R-NC* + *KFDi.n* immunized mice were also observed, though with less statistical significance (Fig. 3f, g). Interestingly, the co-localization of IL-17 secreting cells with plasma cells, as indicated by the marker CD138^+^, was observed only in the *3R-NC* + *KFDi.n* immunized mice (Fig. 3h). While the RBD-specific IgA response in serum and NLF exhibited a robust positive correlation with the Th17 response in CLN, the RBD-specific IgG responses showed no correlation with Th17 response (Fig. 3i). Taken together, we proposed that the Th17 and Tfh17 biased responses in *3R-NC* + *KFDi.n* immunized mice might be linked to the sustained presence of long-lived plasma cell responses.

### The long-term protection against Omicron infection

**Fig. 4:**
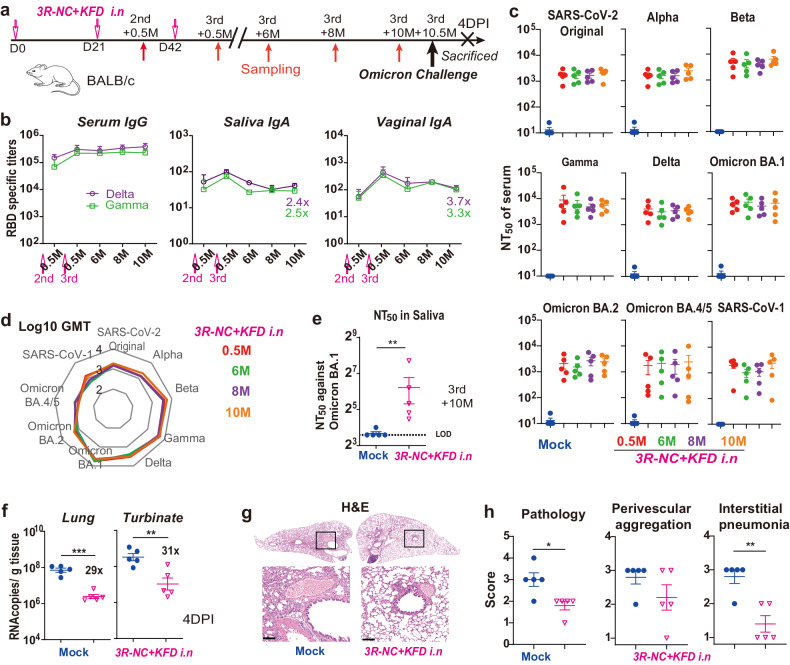
Long-lasting antibody responses and protection against SARS-CoV-2 Omicron BA.1 strain. **a** Diagram scheme of immunization and virus challenge in BALB/c mice (*n* = 5 mice per group). **b** RBD-specific serum IgG, salivary IgA and vaginal IgA at indicated time point post immunization. **c** Neutralization antibody titers against pseudo-typed SARS-CoV-2 variants and SARS-CoV-1, in serum of mock immunized mice (blue) and *3R-NC* + *KFDi.n* immunized mice post 3rd immunization. **d** Geometric mean titers of neutralizing antibody in serum against different pseudo-typed variants at indicated time point post 3rd immunization. **e** Neutralization antibody titers against pseudo-typed Omiron BA.1. **f** qPCR tested RNA copies of SARS-CoV-2 N at 4DPI. **g** Hematoxylin and eosin (H&E) staining of the lung sections (Scale bars, 100 µm). **h** Pathological scores according to the H&E-stained sections. Data are represented as mean ± SEM and are representative of two independent experiments except in (**f**–**h**). Groups were compared using unpaired two-sided Student’s *t* test. **p < 0.01; ***p < 0.001. LOD limit of detection

Then we wondered whether the enduring RBD-specific antibody responses could provide long-term protection against SARS-CoV-2. Previous studies have confirmed the infectivity of SARS-CoV-2 variants in BALB/c mice attributed to the N501Y mutation.^30^ Moreover, compared to hACE2 transgenic models, wild-type BALB/c mice can be readily infected by Omicron BA.1 and exhibited higher levels of replication in the nasal turbinates.^31–33^ Hence, BALB/c mice which can be used for testing vaccine efficacy against SARS-CoV-2,^34^ were selected for intranasally immunized by *3R-NC* + *KFD*, and challenged by an Omicron BA.1 strain at 10.5 months post the 3rd immunization (Fig. 4a). In line with the RBD-specific serum IgG levels (Fig. 4b), neutralizing titers against pseudo-typed-virus of SARS-CoV-2 and SARS-CoV-1 sustained at high level, from 0.5 month to 10 months post the 3rd immunization (Fig. 4c, d). Specifically, the geometric mean titers (GMT) of neutralizing antibodies post 0.5, 6, 8 and 10 months were nearly identical (Fig. 4d), indicating the sustained neutralizing antibody response induced by the *3R-NC* + *KFDi.n* vaccination. Moreover, consistent with the enduring RBD-specific salivary IgA (Fig. 4b), the saliva of *3R-NC* + *KFDi.n* immunized mice still had prominent neutralizing capabilities at 3rd + 10 M (Fig. 4e).

Upon infection with the Omicron BA.1 strain, the *3R-NC* + *KFDi.n* immunized mice showed a significant reduction of viral genome copy numbers in lungs and turbinate tissues, compared to the mock immunized group, by 29-fold and 31-fold respectively (Fig. 4f). Histopathological examination revealed a noticeable decrease in pathology and inflammatory cell infiltration in lung tissues (Fig. 4g, h). These results demonstrated that *3R-NC* + *KFDi.n* not only induced long-lasting neutralizing antibodies, but also offered long-term protection against SARS-CoV-2 variants infection.

### Safety and sequential boost effect of *3R-NC* + *KFDi.n*

To investigate whether *3R-NC* + *KFDi.n* could act as a sequential booster, we initially evaluated the boost effect on hACE2 mice previously primed with inactivated SARS-CoV-2 (IAV) vaccine, a widely used vaccine in China and worldwide (Supplementary Fig. 7a). Similar to that observed in human,^35,36^ RBD-specific serum IgG and neutralizing titers dropped rapidly in the IAV-vaccinated mice (Supplementary Fig. 7b, c). However, two doses of *3R-NC* + *KFDi.n* boosted RBD-specific IgG and neutralizing titers by more than 10 folds (Supplementary Fig. 7d, e).

Encouraged by the results in mice and drawing from extensive experience in developing flagellin-based mucosal vaccines, an investigator-initiated trial was approved (approval number 2022-S090-03) and performed. The objective was to evaluate the safety and immunogenicity of the intranasally administered KFD-adjuvanted 3R-NC vaccine on volunteers who had previously received the IAV two or three times and hadn’t been infected by SARS-CoV-2 before the trial. The primary outcome for safety was the incidence of adverse reactions within 7 days after each dose of inoculation. The primary outcome for immunogenicity was the titer of RBD-specific antibodies post immunization.

Firstly, to identify the Minimum Effect Dose, an open-labeled study with two dosages of 3R-NC plus KFD (25 μg 3R-NC plus 5 μg KFD, 50 μg 3R-NC plus 10 μg KFD) was carried out on four volunteers aged 25- to 35-year-old (Supplementary Fig. 8a). Post immunization of 25 μg 3R-NC plus 5 μg KFD, no adverse effect and no boost effect could be observed (Supplementary Fig. 8b). Post immunization of 50 μg 3R-NC plus 10 μg, while no adverse effect and no boost effect could be observed, the RBD-specific antibody responses and neutralizing antibodies become to be boosted and emerged (Supplementary Fig. 8b, c).

**Fig. 5:**
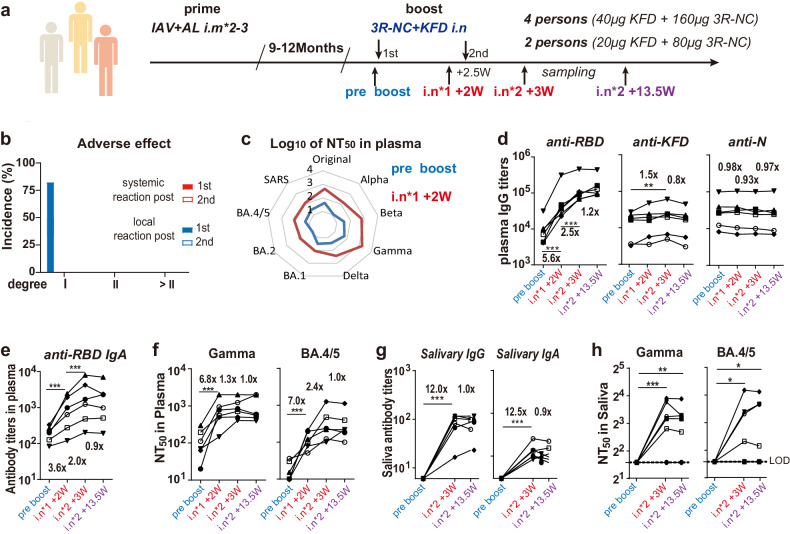
Safety and boost effect of *3R-NC* + *KFDi.n* on IAV primed volunteers. **a** Diagram scheme of the open-labeled pilot study of investigator-initiated trial on IAV vaccinated volunteers. Two persons received lose dose (80 µg 3R-NC plus 20 µg KFD), four persons received high dose (160 µg 3R-NC plus 40 µg KFD). **b** Adverse effects post the 1st and 2nd doses of 3R-NC plus KFD. **c** Neutralization titers against pseudo-typed SARS-CoV-2 variants and SARS-CoV-1, in plasma of one person pre- and post- the 1st dose of boost. **d** RBD, N and KFD1 specific IgG titers in plasma. **e** RBD-specific IgA titers in plasma. **f** Neutralizing titers in plasma against pseudo-typed SARS-CoV-2 Gamma strain and Omicron BA.4/5 strains. **g** RBD-specific IgG and IgA titers in saliva. **h** Neutralizing titers in saliva against pseudo-typed SARS-CoV-2 Gamma strain and BA.4/5 strains. In (**d**–**f**), different individuals were indicated by different symbols. Data are represented as mean ± SEM. Groups were compared using one-way ANOVA. *p < 0.05; **p < 0.01; ***p < 0.001. LOD limit of detection

Next, six volunteers aged 20- to 30-year-old were intranasally immunized with two doses of KFD-adjuvanted 3R-NC, with either 80 μg 3R-NC plus 20 μg KFD (2 persons) or 160 μg 3R-NC plus 40 μg KFD (4 persons) at the end of December 2022 (Fig. 5a). The trial results showed that adverse reactions after immunization were mild (grade I), observed only after the 1st dose, and no systemic adverse reactions occurred (Fig. 5b and Supplementary Table 1). The adverse reactions were limited to the nose area (Supplementary Fig. 9), appeared at 1–4 h post immunization, and vanished within 12 h post immunization.

In human subjects, a single dose of *3R-NC* + *KFDi.n* can significantly boost neutralizing antibody responses against SARS-CoV-2 VOCs and SARS-CoV-1 (Fig. 5c, and left panel of 5d). Subsequently, the second dose of *3R-NC* + *KFDi.n* provided an additional 2.5-fold boost to RBD-specific IgG responses (left panel of Fig. 5d). In contrast to RBD-specific response, the boost effect on KFD-specific IgG was minimal (middle panel of Fig. 5d). Notably, from the end of December 2022 to April 2023, none of the six volunteers experienced respiratory infection symptoms, despite a significant wave of SARS-CoV-2 spread in China from the end of December 2022 to February 2023. To address concerns that these individuals may have been inadvertently infected SARS-CoV-2 during the study period, either without experiencing symptoms or being asymptomatic, tests were conducted at various time points to detect antibodies against the SARS-CoV-2 N protein. The consistent presence of N protein-specific IgG responses in plasma further indicated that the boosted RBD-specific antibody responses were not induced by infection (right panel of Fig. 5d).

Consistent with the findings regarding RBD-specific IgG antibodies, the boost effect on RBD-specific IgA antibodies and neutralizing antibodies in plasma was prominent, resulting in approximate a 10-fold elevation (Fig. 5e, f). Notably, although 3R-NC only contains RBDs of Gamma and Delta strains, *3R-NC* + *KFDi.n* could also boost neutralizing antibodies against the Omicron BA.4/5 in plasma (Fig. 5f). In saliva samples, RBD-specific IgG antibodies, IgA antibodies, and neutralizing ability all showed significantly increases post boost (Fig. 5g, h), same as that in plasma. Moreover, from 3-week to 13.5-week post the second dose, RBD-specific antibodies and neutralizing titers in both plasma and saliva displayed no signs of reduction (Fig. 5d–h). These results strongly indicated that *3R-NC* + *KFDi.n* can effectively act as a sequential booster in humans, enhancing immune responses and providing potential long-lasting protection against the SARS-CoV-2 variants.

### Adaptable triple-RBD scaffold protein: readily modifiable for emerging SARS-CoV-2 variants

**Fig. 6:**
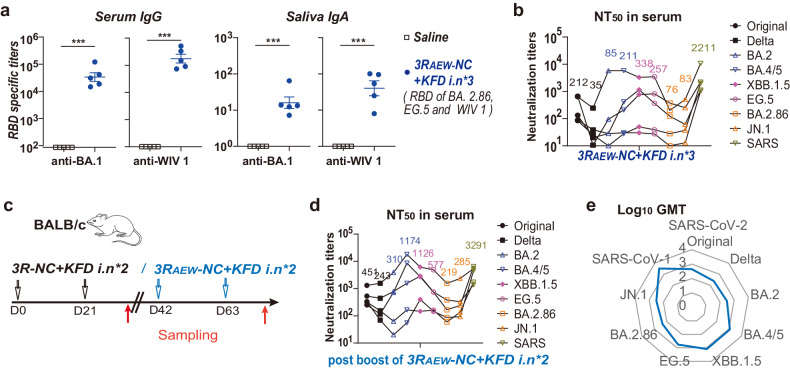
The antibody responses and neutralizing titers induced by *3R*_*AEW*_*-NC* + *KFDi.n* without or with *3R-NC* + *KFDi.n* prime. **a**, **b** BALB/c mice were intranasally immunization 3 doses of *3R*_*AEW*_*-NC* + *KFDi.n* or Saline (*n* = 5 mice per group). Serum and saliva were sampled at 2 weeks point post 3rd immunization. **a** RBD-specific serum IgG and salivary IgA post 3rd immunization. **b** Neutralizing antibody titers against pseudo-typed SARS-CoV-2 variants and SARS-CoV-1 in serum of *3R*_*AEW*_*-NC* + *KFDi.n* immunized mice post 3rd immunization. **c** Diagram scheme of the immunization and sampling of 2 doses of *3R*_*AEW*_*-NC* + *KFDi.n* boosts on the *3R-NC* + *KFDi.n* primed BALB/c mice (*n* = 5 mice per group). **d**, **e** Neutralization antibody titers against pseudo-typed SARS-CoV-2 variants and SARS-CoV-1 in serum of immunized mice post boost. Data are represented as mean ± SEM which were compared with unpaired two-sided Student’s *t* test. ***p < 0.001

Previous RBD-scaffold immunogen designs have shown success in eliciting long-lasting immune responses and offering broad-spectrum protection. To validate our triple-RBD scaffold platform’s adaptability to circulating SARS-CoV-2 strains, we developed 3R_AEW_-NC, incorporating RBDs from BA.2.86, EG.5, and SARS-like WIV 1. In mice, intranasal administration of 3R_AEW_-NC with flagellin KFD (*3R*_*AEW*_*-NC* + *KFDi.n*) effectively induced RBD-specific serum IgG against SARS-CoV-2 and WIV 1, accompanied by a salivary IgA response after the third dose (Fig. 6a). Notably, moderate neutralization efficacy against emerging BA.2.86 lineages (BA.2.86 and JN.1) were displayed (Fig. 6b and Supplementary Fig. 10). Furthermore, upon boosting *3R-NC* + *KFDi.n* pre-immunized mice with *3R*_*AEW*_*-NC* + *KFDi.n* (Fig. 6c and Supplementary Fig. 11), a noticeable enhancement of neutralizing activity against BA.4/5, XBB.1.5 and particularly BA.2.86 lineage strains (BA.2.86 and JN.1) was evident (Fig. 6d). Importantly, this strategy generated broad-spectrum neutralizing antibodies targeting diverse SARS-CoV-2 variants, including the XBB lineage, BA.2.86 lineage, and even SARS-CoV-1 (Fig. 6d). These findings highlight the inherent adaptability of the RBD-based scaffold design, allowing readily antigenic updates and holding promise for establishing durable immunity.

## Discussion

In the pursuit of ideal SARS-CoV-2 vaccines, we should face three hurdles: inducing broad protection against rapidly emerging variants, establishing mucosal immunity, and ensuring persistent neutralizing antibodies. Among these challenges, the generating of long-lasting neutralizing antibodies has been proven to be the most formidable task. For developing subunit mucosal vaccines, the lack of safe and effective mucosal adjuvant for human is another obstacle.

Multivalent approaches have been reported to increase BCR cross-linking, improve transporting antigens into the B cell follicles, and enhance cross-neutralization.^37,38^ In this study, we successfully developed a triple-RBD scaffold protein (3R-NC) which shows promising stability and antigenicity while preserving crucial neutralizing epitopes (Fig. 1). Compared to RBD monomer, 3R-NC exhibited higher efficiency being taken up by DCs and binding to RBD-specific B cells, in addition to significantly higher antigenicity (Fig. 1 and Supplementary Fig. 2). Multivalent are high stability were proposed to promote the generation of long-term humoral immunity.^39,40^ When administered intranasally with the flagellin-derived adjuvant KFD (*3R-NC* + *KFDi.n*), high levels of RBD-specific cross-protective antibodies against SARS-CoV-2 variants were generated, in both serum and mucosal sites (Fig. 2 and Supplementary Fig. 4).

It’s worth noting that these RBD-specific neutralizing antibodies demonstrated remarkable durability, persisting for more than 13 months in mice, particularly in the group of *3R-NC* + *KFDi.n* (Fig. 2d–g). LLPCs are known to be crucial for maintaining long-term humoral immunity,^39^ and can exist in mucosal sites.^41^ Further analysis revealed that *3R-NC* + *KFDi.n* induced a substantial number of LLPCs (Fig. 2h–m), especially high amount of RBD-specific IgA-secreting LLPCs in nose (Fig. 2h). The co-expression of J chain (Supplementary Fig. 4) further suggested that most of these LLPCs in the nose consistently secreting dimmer or polymer IgA. However, it remains to be determined whether these RBD-specific IgA-secreting cells were circulated from the bone marrow or long-term existed in the nasal mucosa.

Indeed, previous research has emphasized the critical role of Tfh in regulating the differentiation of GC B cells and the development of long-term humoral immunity. Induction of LLPCs by Tfh cells particularly relies on the production of IL-21.^42,43^ The observed upregulation of IL-21 secretion in the *3R-NC* + *KFDi.n* group was closely associated with high levels of LLPCs (Fig. 3), indicating the vaccine’s potential in promoting long-lasting protective immune responses. Furthermore, in the induction of IL-21 and the generation of LLPCs, IL-12 and IL-6 were reported to the most potent inducers.^42,44,45^ Therefore, the IL-6 and IL-12 induced by intranasal administration of flagellin,^29,46^ likely play a critical role in shaping the Tfh response and promoting the development of LLPCs induced by *3R-NC* + *KFDi.n*.

In *3R-NC* + *KFDi.n* immunized mice, the Teff and Tfh responses induced by the immunization displayed a Th17 biased feature (Fig. 3). Previous research has underscored the pivotal role of IL-17 and Tfh17 in GC B cell activation,^47^ as well as the generation and sustainability of LLPCs, particularly in systemic lupus erythematosus (SLE).^48–50^ Moreover, compared to Tfh1 and Tfh2 cells, Tfh17 cells have a stronger tendency to induce LLPCs generation.^51^ Interestingly, similar Th17 biased CD4^+^ T cell responses have been observed in another flagellin-based mucosal vaccine, P-KFD1.^23^ The co-localization of IL-17 with the plasma marker CD138 (Fig. 3) suggested a link between the vaccine-induced LLPCs and the Th17 biased responses. Nonetheless, the Th17 response did not correlate with RBD-specific IgG induction. Our previous work confirmed that TLR5 activation by flagellin induces GM-CSF secretion by epithelial cells, crucial for mucosal adjuvanticity and antigen-specific IgA generation.^29^ It is reasonable to propose that the Th17 and Tfh17-biased responses might not be critical for the induction of RBD-specific antibody responses but rather essential for the generation and sustainability of LLPCs in the context of flagellin-based mucosal vaccines. A small open-labeled investigator-initiated trial in humans suggested that *3R-NC* + *KFDi.n* successfully boosted RBD-specific neutralizing antibody responses and demonstrated impressive safety and tolerance (Fig. 5 and Supplementary Figs. 8 and 9). Despite *3R-NC* only contains RBDs form Gamma and Delta strains, the neutralizing titers against BA.4/5 strain can be boosted more than 10 times in plasma and in saliva. This can help the volunteers resist the large wave of SARS-CoV-2 strains BA.5.2 and BF.7 that struck China from December 2022 to February 2023. While the trial’s limitations include the absence of a control group and its relatively small scale, it nonetheless provided preliminary evidence of the safety and immunogenicity of *3R-NC* + *KFDi.n*. Notably, this pilot study still indicated the safety and effectiveness of the flagellin based mucosal adjuvant KFD. Further research and larger clinical trials will be necessary to validate the efficacy and safety of mucosal adjuvant KFD and the KFD adjuvanted triple-RBD based SARS-CoV-2 mucosal vaccine on a broader scale. Due to the small sample size and the involvement of only 20–30-year-old healthy volunteers in the study, a more meticulous evaluation of systemic and local inflammatory responses and safety profiles by this adjuvant should be conducted before large-scale human trials.

Our findings underscore the significant potential of *3Rx-NC* + *KFDi.n* as a prototype of subunit mucosal vaccines against SARS-CoV-2. In both animal and human studies, it effectively addressed three key challenges: inducing mucosal responses at the site of first viral contact, protecting against a wide range of viral variants, and generating long-lasting neutralizing antibodies. Importantly, the RBD-scaffold design allows for rapid adaptation to new variants, making it versatile for developing potent mucosal vaccines not only against COVID-19, but also other emerging infectious threats.

## Material and methods

### Animals

The 5–7 weeks old female BALB/c mice were purchased from Beijing Vital River Laboratory Animal Technology Company. After randomly assigned, all groups of mice were raised under specific pathogen-free (SPF) conditions in individually ventilated cages (IVCs).

### Protein and vaccine preparation

The inactivated virus (IAV) of original strain SARS-CoV-2, the recombinant proteins KFD, 3R-NC, 3R_AEW_-NC and RBDs were generated and purified as previously described^26,28^ Briefly, the KFD gene was constructed by connecting the N-terminal and C-terminal regions of the D0-D1 domains of flagellin KF (*E. coli* K12 strain MG1655), cloned into the pET-28a plasmid vector with his-tag in C-terminal domain (Invitrogen), and transformed into the *E. coli* BL21 DE3 strain. The 3R-NC, 3R_AEW_-NC and RBDs (aa. 319–527) were respectively cloned into the pcDNA3.1 vector with the presence of signal peptide tPA in the 5’ region and his-tag in C-terminal domain. The recombinant plasmids were transfected with polyethyleneimine (PEI) into HEK293F cells (Thermo Fisher Scientific).

These recombinant proteins were purified by affinity chromatography on a Ni-NTA column (QIAGEN), then over a Superdex 200 Increase 10/300 GL column (GE Life Sciences). Finally, after sterile filtration, the endotoxin removal kit (YEASEN) was applied to remove the contaminating lipopolysaccharide (LPS). According to the Limulus assay (BIOENDO), residual LPS content were measured below 0.02 EU/μg protein.

### Mice immunization and challenge

After pentobarbital sodium anesthesia, the 6–8 weeks old female BALB/c mice were intramuscularly immunized at lower limb with 4 μg 3R-NC plus 200 μg Alum adjuvant (Imject^TM^, Thermo Fisher) in 50 ul PBS, or intranasally immunized with 1 μg KFD plus 4 μg 3R-NC or 3R_AEW_-NC in 10 ul PBS at 3-week intervals. For challenge experiments on BALB/c mice, mice were intranasally inoculated with 4.35 × 10^4^ TCID_50_ of Omicron strain BA.1 (BA1-HB0000428) in 50 μl under avertin (250 mg/kg) anesthesia. At 4 dpi, after euthanized, the lung and turbinate tissues of mice were harvested.

### Human experiments

Human experiment was performed on healthy volunteers aged between 20 and 30 years old, who had vaccinated with 2 or 3 doses of the inactivated original strain SARS-CoV-2 vaccine (BBIBP-CorV). These volunteers were immunized intranasally with 80 μg 3R-NC plus 20 μg KFD (2 persons) or 160 μg 3R-NC plus 40 μg KFD (4 persons) in 200 μl PBS at 20-day intervals. Briefly, 100 μl vaccine was sprayed into one side of nose by nasal sprayer (Nest), and another 100 μl was sprayed into the other side of nose.

### Pseudo-typed virus preparation and neutralization assay

As described previously, the plasmid of Env-defective HIV-1 (pNL4-3.luc.RE) and plasmid expressing spike of SARS-CoV-2 or SARS-CoV-1 were co-transfected into HEK293T cells to generate pseudovirus. To test neutralization capacity, heat-inactivated samples were serially diluted three or four times with complete medium in a volume of 50 μl. Next, 20 μl of 200 50% tissue culture infectious doses (TCID_50_) of the SARS-CoV-2 pseudovirus were mixed with the diluted samples and co-incubated at 37 °C. One hour later, pre-prepared 3 × 10^5^ ACE2-expressing HEK293 cells of 30 μl in DMEM were added to 96-well plate. After 48 h, luciferase activity of the cells was measured by the luciferase assay system (Promega). The 50% neutralization titers (NT_50_) were determined by a four-parameter logistic regression using GraphPad Prism as previously described.^27^

### Immunocytes isolation

For isolation of nasal immunocytes, mice noses were cut into pieces around 2 mm × 2 mm, and digested by 2 mg/ml collagenase IV plus 50 μg/ml DNase I (Sigma-Aldrich) in 2 ml RPMI 1640 at 37 °C for 30 min. After pressed through a 70 μm nylon mesh screen and washed by PBS, the cell pellets were resuspended in 40% Percoll, layered onto 70% Percoll (GE Healthcare). After centrifugation at 2000 rpm for 25 min, cells in the interface between 40% and 70% Percoll gradients were aspirated, and then washed by PBS.

For isolation of immunocytes in CLNs, mice CLNs were digested by 1 mg/ml collagenase D and 50 μg/ml DNase I (Sigma-Aldrich) in 1 ml RPMI 1640, at 37 °C for 30 min. After pressed through a 70 μm nylon mesh screen, single-cell suspensions were collected and washed by cold PBS.

For isolation of splenic lymphocytes, mice spleens were minced. After pressed through 70 μm nylon mesh screen, using Mouse RBC Lysis Buffer (Dakewe), and washed by PBS, splenic lymphocytes were obtained.

### Flow cytometry

RBD and 3R-NC were labeled with AF647 according to the guidance of Protein Labeling Kits for Alexa Fluor™647 (cat. no. A20173, Thermo Fisher). The concentration of AF647 labeled protein and the AF647 labeling efficiency were assayed by Nanodrop one (Thermo Fisher) in the first label dye mode. To detect the antigen presentation by DCs or antigen-specific B cells uptakes in vitro, splenic lymphocytes were incubated with 3R-NC-AF647 (1 μg/ml) or RBD-AF647 (1 μg/ml) for 3 h at 37 °C. After blocking nonspecific Fc receptor binding by anti-CD16/CD32 (93, cat. no. 101301, BioLegend), the cells were stained with the following: PB anti-CD11c (N418, cat. no. 117322, BioLegend), APC/Cy7 anti-I-A/I-E (MHCII) (M5/114.15.2, cat. no. 107628, BioLegend), BV785 anti-B220 (RA3-6B2, cat. no. 103246, BioLegend) and fixable viability dye (cat. no. 65-2860-40, eBioscience) at 4 °C for 30 min.

For assay of Tfh response, single cells suspension was stimulated at 37 °C for 5 h by cell activation cocktail with brefeldin A and monensin. After labeled with PB anti-CD4 (RM4-5, cat. no. 100534, BioLegend), AF700 anti-CD3 (17A2, cat. no. 100216, BioLegend), APC/Cy7 anti-CD44 (IM7, cat. no. 103028, BioLegend), PE anti-CXCR5 (L138D7, cat. no. 145504, BioLegend), PE/Dazzle™ 594 anti-PD-1 (29F.1A12, cat. no. 135228, BioLegend) and fixable viability dye, cells were fixed by fixation buffer, permeabilized by Intracellular Staining Permeabilization Wash Buffer, and then stained intracellularly with mAbs specific to APC anti-IL-21 (FFA21, cat. no. 17-7211-80, eBioscience), FITC anti-IFN-γ (XMG1.2, cat. no. 505805, BioLegend) and PerCP/Cyanine5.5 anti-IL-17A (TC11-18H10.1, cat. no. 506919, BioLegend).

LSRFortessa (BD, Heidelberg, Germany) and FlowJo software (Tree Star, Ashland, OR) were used to collect and analyze data.

### ELISPOT

For simultaneous detection IFN-γ, IL-17A and IL-5 secreting cells, the Mouse IFN-γ/IL-17A/IL-5 FluoroSpot kit (cat. no. FSP-414443-2, Mabtech) was used. Briefly, 5 × 10^5^ of lymphocytes from spleen or cervical lymph nodes were seeded into the pre-coated FluoroSpot plate and stimulated with SARS-CoV-2 S-RBD peptides pool (0.5 μg/ml, JPT PM-WCPV-S-1) at 37 °C, with 5% CO_2_ for 42 h. The cell culture detection mAbs mix, Fluorophore conjugates mix, and FluoroSpot enhancer were sequentially added. Numbers of spots were counted by automatic FluoroSpot Reader (mabtech IRIS, Sweden).

For detection of RBD-specific IFN-γ secreting CD4^+^ T cells, splenic CD4^+^ T cells were positive isolated by magnetic beads (cat. no. 130-117-043, Miltenyi Biotec). Then 1.25 × 10^5^ of positive selected CD4^+^ T cells were mixed with 7.5 × 10^5^ naïve splenocyte as antigen presenting cells and stimulated by SARS-CoV-2 S-RBD peptide (0.5 μg/ml) or RPMI 1640 in 96-well flat-bottom plates (Millipore) that pre-coated with anti-mouse IFN-γ coating antibody (cat. no. CT655, U-CyTech). After culturing at 37 °C with 5% CO_2_ for 42 h, biotin-conjugated mAbs and streptavidin-HRP were sequentially added, and then the AEC coloring system (cat. no. 2030613, DAKEWE) was used to develop spots as previously described.^23^

Before detection of RBD-specific IgG or IgA antibody secreting cells (ASCs), RBD was labeled with Biotin (Thermo Fisher) and stored in −80 °C. The lymphocytes cells from spleen, nose or bone marrow were resuspended, added into the goat anti-mouse IgG-UNLB (cat. no. 0107-01, SouthernBiotech) or goat anti-mouse IgA-UNLB (S107, cat. no. 0106-01, SouthernBiotech) precoated ELISpot plate (Millipore), and then incubated at 37 °C for 18 h with 5% CO_2_. After washing plates, RBD-biotin and streptavidin-HRP were applied sequentially. At last, spots were developed by AEC coloring system, and counted by automatic EliSpot System Classic (AID, Germany).

### Histology of lung

After fixed in 4% paraformaldehyde for 7 days, lung tissues were embedded in paraffin, cut into slices. After hematoxylin and eosin (H&E) staining, pathological changes were evaluate and scored on a 0–5 severity scale, according to the thickened alveolar walls, cell aggregation, blocked bronchioles and lung consolidation. Infiltration was also evaluated and scored on a 1–4 severity scale.

For immuno-fluorescence assay, the sections were deparaffinized and rehydrated, followed by Citrate-mediated antigen retrieval. After blocking, the primary antibody Anti-Syndecan-1/CD138 Rabbit pAb (cat. no. GB115052, Servicebio), HRP-conjugated goat-anti-rabbit IgG and CY3-Tyramide (cat. no. G1223, Servicebio) were used, followed by Citrate-mediated antigen retrieval. Then another primary antibody Anti-IL-17 Rabbit pAb (cat. no. GB11110-1, Servicebio), secondary antibody HRP-conjugated goat-anti-rabbit IgG and the corresponding iF488-Tyramide (cat. no. G1231, Servicebio) were used. Cell nuclei were stained using 49, 6-diamino-2-phenylindole (DAPI) (cat. no. G1012, Servicebio). The slide scanner Pannoramic MIDI (3DHISTECH) was utilized to image whole slide.

### Statistical analysis

The GraphPad Prism 8.0 software were used to perform analyses. Statistical analysis was performed using one-way ANOVA, then followed by Dunnett’s multiple comparison test, except otherwise indicated. To analyze the differences between two groups, for normally distributed data with homogeneous variance, unpaired two-tailed Student’s *t* test was used. For non-normally distributed data the Mann–Whitney *U* test was used. The simple linear regression method was used for correlation analysis. *p* < 0.05 was considered significant. The significance values are indicated as: ***p < 0.001, **p < 0.01, *p < 0.05 and ns non-significant.

### Supplementary information

revised Supplements clean version

## Data Availability

All data are present in the paper or in Supplementary Materials. Upon request, additional information required to reanalyze the data is available from the lead contact.
