# Supplementary material for: Nasal vaccination of triple-RBD scaffold protein with flagellin elicits long-term protection against SARS-CoV-2 variants including JN.1
Source: Signal Transduct Target Ther. 2024 Apr 27;9:114. doi: 10.1038/s41392-024-01822-3 (PMC11055866; doi:10.1038/s41392-024-01822-3)
Supplement: Supplementary file 1 — revised Supplements clean version [file 41392_2024_1822_MOESM1_ESM.pdf]

Supplementary Materials for

Nasal vaccination of triple-RBD scaffold protein with flagellin elicits long-term protection against SARS-CoV-2 variants including JN.1

Xian Li*, Mengxin Xu*, Jingyi Yang*,†, Li Zhou, Lin Liu, Min Li, Shasha Wang, Mei-Qin Liu, Zhixiang Huang, Zhen Zhang, Shuning Liu, Yunqi Hu, Haofeng Lin, Bowen Liu, Ying Sun, Qingguo Wu, Zheng-Li Shi, Ke Lan, Yu Chen†, Huimin Yan†, Yao-Qing Chen†

Correspondence to: chenyaoqing@mail.sysu.edu.cn (Y-Q.C.); yanhuimin@shphc.org.cn (H.Y.); chenyu@whu.edu.cn (Y.C.); yangjingyi@shphc.org.cn (J.Y.)

This PDF file includes:

Materials and Methods
Figures S1 to S11
Table S1

Materials and Methods

Samples collection and antibody measurement

Mice sera and mucosal secretions were collected after immunization for analysis of RBD or flagellin-specific antibody responses as previously described.[1, 2] Briefly, vaginal lavage fluid was collected by aspirating 40μL of PBS into the vaginal tract with 200μL tips. The fluid was blown and aspirated 10 times, washed twice and mixed together. Saliva was procured after carbamylcholine (Sigma) intraperitoneal injection. Serum was obtained by collecting blood samples from the retro-orbital plexus after anesthesia. For collection of nasal washes and bronchoalveolar lavage fluid (BALF), the trachea was exposed surgically post sacrificed the mice. After a small cut was made on the trachea with small surgical scissors, a 1-ml tip covered with a 10-μl tip containing 300-μl PBS was carefully inserted into the trachea with tip toward nose and washed two times. Next, a 1-ml tip covered with a 10-μl tip containing 600-μl PBS was carefully inserted into the trachea with tip toward lung and pipetted eight times. For human, plasma was collected from blood of the cubital vein. Saliva was collected without stimulation. The mucosal samples were centrifuged at 4000 rpm for 10 minutes before being stored. All samples were stored at -80°C until they were assayed by ELISA. Alkaline phosphatase-labeled goat anti-mouse IgG, IgA, IgG1 or IgG2a (SouthernBiotech) polyclonal antibodies and substrate (p-nitrophenyl phosphate; Sigma) were used for detection.

Flow cytometry

For detection the generation of B cells and plasma cells, the single-cell suspensions of splenic cells were labeled with fixable viability dye and surfaced-stained with mAbs specific to BV785 anti-B220 (RA3-6B2, cat. no. 103246, BioLegend), BV421 anti-CD138 (281-2, cat. no. 142523, BioLegend), PerCP/Cyanine5.5 anti-GL7 (GL7, cat. no. 144610, BioLegend), PE/Dazzle 594 anti-CD38 (90, cat. no. 102729, BioLegend), RBD-AF647 and PNA (biotin conjugate) (cat. no. L6135, Sigma-Aldrich®) with FITC Streptavidin (cat. no. 405201, BioLegend).

For detection the DCs activation of CLNs and nose in the immunized mice, naïve BALB/c mice were intranasally immunized by 8 μg 3R-NC-AF647 with 2 μg KFD. After 18h, CLNs and nose immunocytes were isolated. Then the single-cell suspensions were labeled with fixable viability dye and surfaced-stained with mAbs specific to PB anti-CD11c (N418, cat. no. 117322, BioLegend), APC-Cy7 anti-I-A/I-E (MHCⅡ) (M5/114.15.2, cat. no. 107627, BioLegend), PE anti-CD197 (CCR7) (4B12, cat. no. 120106, BioLegend) and AF488 anti-CD86 (GL-1, cat. no. 105017, BioLegend).

ELISPOT

For detection of total IgA secreting cells, ELISpot plate (Millipore) wells of plate were coated by the goat anti-mouse IgA-UNLB (S107, cat. no. 0106-01, SouthernBiotech) to capture the IgA secretion by the lymphocytes cells from nose or bone marrow. After 18 hours of cell culturing, cells were removed. AP labeled goat anti-mouse IgA (S107, cat. no. 0106-01, SouthernBiotech) was incubated. Then the spots (blue-purple) were developed with the BCIP/NBT Alkaline Phosphatase Color Development Kit (cat. no.

C3206, beyotime).

As for the detection of RBD-specific IgA antibody secreting cells types, ELISpot plate (Millipore) wells of plate were coated by the goat anti-mouse IgA-UNLB (S107, cat. no. 0106-01, SouthernBiotech) to capture the IgA secretion by the lymphocytes cells from nose or bone marrow. After 18 hours of cell culturing, cells were removed. The wells were incubated with rabbit anti-J chain polyclonal antibody (cat. no. FNab04187, FineTest) and biotin labeled RBD. Then AP conjugated goat anti-Rabbit IgG (cat. no. SA00002-2, Proteintech Group) and HPR labeled streptavidin were incubated. Next, the BCIP/NBT Alkaline Phosphatase Color Development Kit were used to staining the J chain expressed IgA secreting cells (blue-purple). After 15 minutes, the reaction was stopped by gently rinsing membrane with tap water by gently rinsing membrane with tap water. At last, the AEC Coloring System (cat. no. 2030613, DAKEWE) were used to staining the RBD-specific IgA secreting cells (red-brown). The co-localizations were red spots with dark brown- black center.

The hACE2 mice study

The HFH4-hACE2 transgenic mice on C57BL/6 background (from Dr. Ralph Baric of the University of North Carolina) were bred and housed at the Animal Center of WIV, CAS. The infection experiments on HFH4-hACE2 transgenic mice were performed in the Animal Biosafety Level 3 (ABSL-3) Laboratory of WIV, CAS.

For challenge experiments on hACE2 mice, under avertin anesthesia, the mice were intranasally inoculated with 3×10^4 $TCID_{50}$ of Omicron strain BA.1 (IVCAS6.7600) in 30 μL. At 3 dpi, after euthanized, the lung and turbinate tissues of

mice were harvested.

References

1. Zhang, Y. et al. Unpolarized release of vaccinia virus and HIV antigen by colchicine treatment enhances intranasal HIV antigen expression and mucosal humoral responses. *PLoS One* **6**, e24296 (2011).
2. Yang, J. et al. Antigen replacement of domains D2 and D3 in flagellin promotes mucosal IgA production and attenuates flagellin-induced inflammatory response after intranasal immunization. *Hum Vaccin Immunother* **9**, 1084-1092 (2013).
3. Yang, J. et al. Second-generation Flagellin-rPAc Fusion Protein, KFD2-rPAc, Shows High Protective Efficacy against Dental Caries with Low Potential Side Effects. *Sci Rep* **7**, 11191 (2017).

Supplementary figures

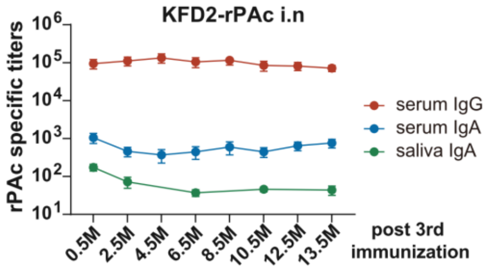

Fig. S1. The rPAC specific antibody responses post the 3rd immunization of KFD2-rPAc. 6-8 weeks old female BALB/c mice were intranasally administrated 3 times at 4-week intervals with 1.4 μg of flagellin based caries vaccine KFD2-rPAc in 10μl PBS as previously described.[3] The serum and saliva collected at indicated time points post the 3rd immunization were detected by ELISA for antibody response against rPAc. Data are represented as mean ± SEM.

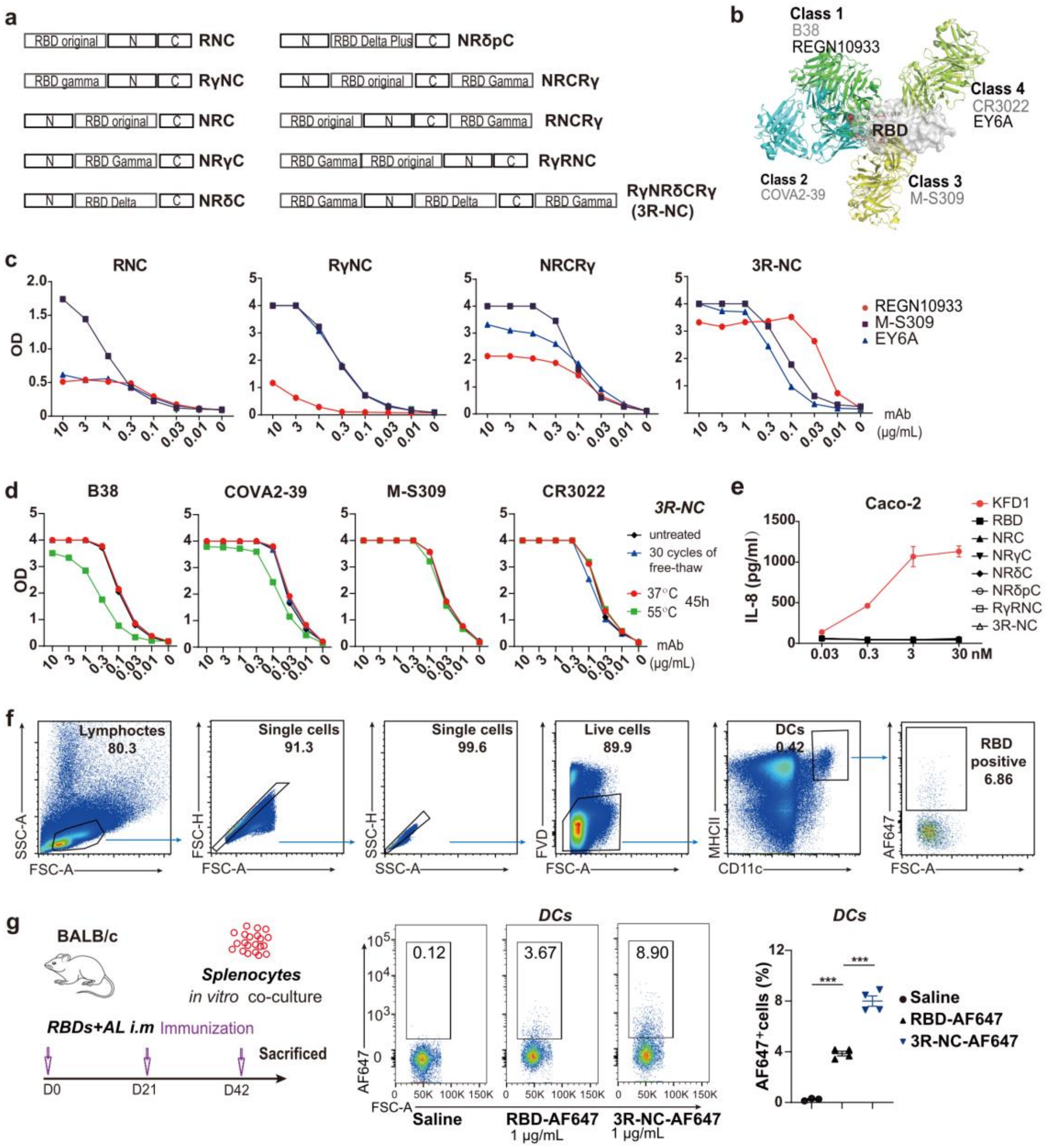

Fig. S2. Characteristics of RBD based recombinant proteins. Related with Fig. 1. (**a**) Diagram of the RBD based recombinant proteins. (**b**) Diagram of the binding of SARS-CoV-2 RBD with four classes of neutralizing mAbs. (**c**) ELISA analyzed binding ability of these RBD based recombinant proteins with four classes of representative neutralizing mAbs (n=3). (**d**) The stability of purified protein 3R-NC in resistance to high temperature and freeze-thaw cycles, tested by the binding ability with representative neutralizing mAbs by ELISA. (**e**) TLR5 agonist efficacy tested by ELISA assayed IL-8 secretion from the recombinant proteins stimulated Caco-2 cells (n=3). (**f**) Gating strategy of DCs (MHCII^{+} CD11c^{+}) and AF-647 positive DCs. (**g**) The uptake of the AF-647 labled RBD or 3R-NC by splenic DCs from RBD immunized mice (n=4 mice per group). Data are represented as mean ± SEM and are representative of two

independent experiments. Groups were compared using one-way ANOVA. $^{**}P < 0.01$; $^{***}P < 0.001$; ns, nonsignificant.

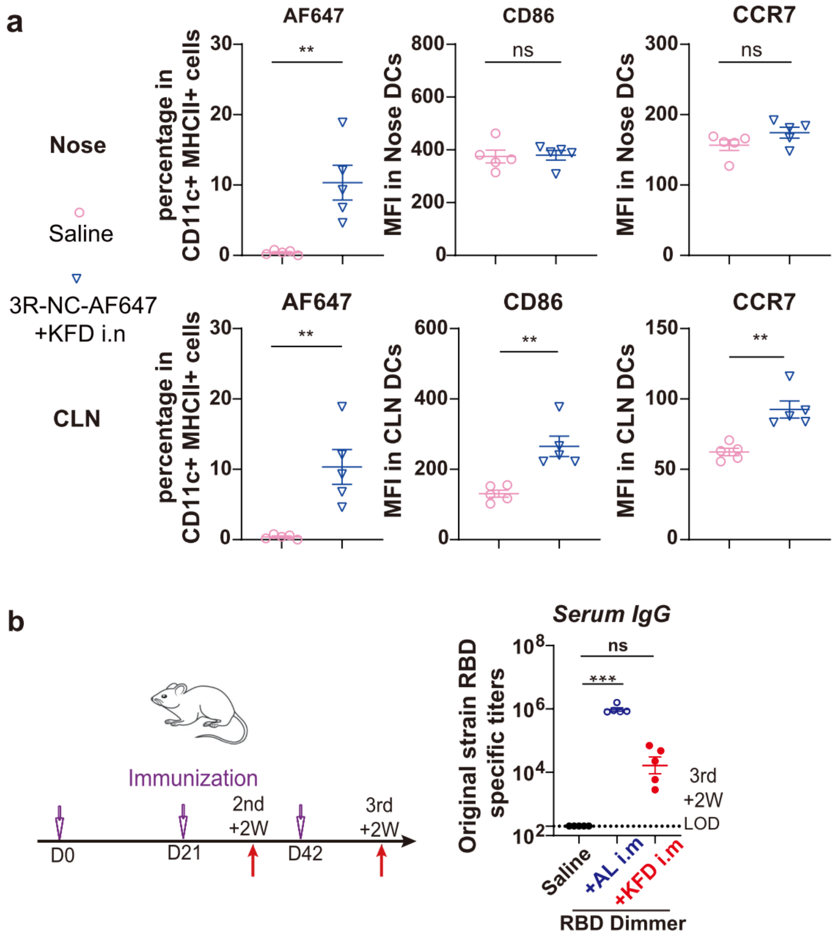

Fig. S3. The adjuvanticity of KFD via intranasal or intramuscular administration. (**a**) *3R-NC-AF647+KFDi.n* (8μg 3R-NC-AF647 plus 2 μg KFD) was intranasally administrated and DC activation was assay at 18 hours post administration (n=5 BALB/c mice per group). (**b**) RBD-specific IgG in serum of mice responses post the 3rd i.m immunization of *RBD dimmer+ALi.m* (4μg original strain RBD dimmer plus 100 μg AL adjuvant) and *RBD dimmer +KFDi.m* (4μg original strain RBD dimmer plus 1 μg KFD) (n=5 hACE2 mice per group). Data are represented as mean ± SEM. Groups were compared using one-way ANOVA. ***P* < 0.01; ****P* < 0.001; ns, nonsignificant.

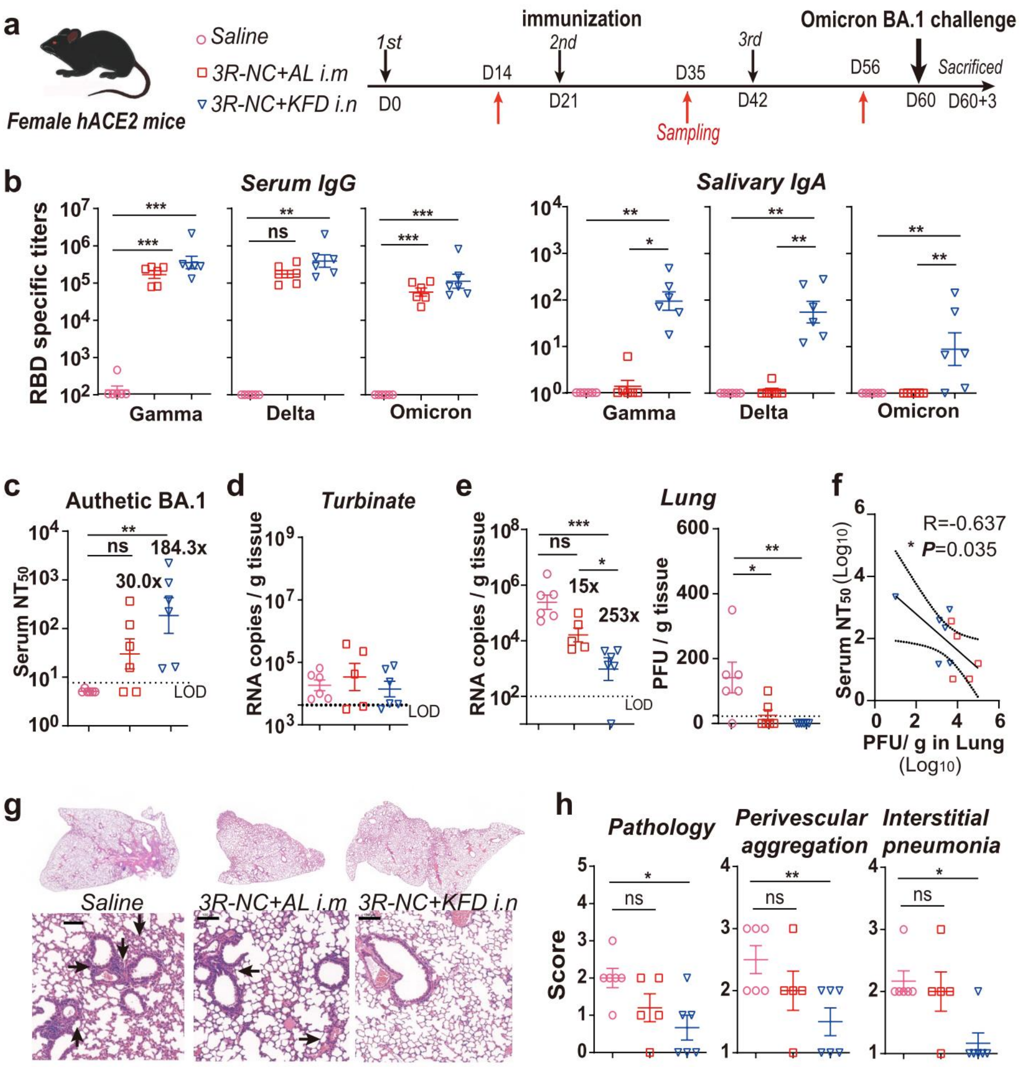

Fig. S4. Protection in the immunized hACE2 mice against SARS-CoV-2 Omicron BA.1 strain infection. Female hACE2 mice were immunized and challenged by Omicron BA.1 strain (IVCAS6.7600) (n = 6 mice per group, one mouse in the group of *3R-NC+ALi.m* dead by accident in the challenge procedure). (**a**) Diagram scheme of immunization and virus challenge. (**b)** RBD-specific serum IgG and salivary IgA post the 3rd immunization. (**c)** Neutralization antibody titers in serum against authentic SARS-CoV-2 Omicron BA.1 strain post the 3rd immunization. (**d** and **e**) qPCR tested RNA copies of SARS-CoV-2 RBD and plaque assay tested infectious virus in lung and turbinate tissue at 3DPI. (**f**) Correlation of qPCR tested RNA copies with the NT_{50} against authentic Omicron BA.1 in serum. The 95% confidence interval was indicated by dotted lines. (**g**) Hematoxylin and eosin (H&E) staining of the lung sections (Scale bars, 100 µm; Arrow, immunocyte infiltration). (**h**) Pathological scores according to the H&E-stained sections. Groups were compared using one-way ANOVA

except in (f), which was compared using simple linear correlation. *$P < 0.05$; **$P < 0.01$; ***$P < 0.001$; ns, nonsignificant. LOD, limit of detection.

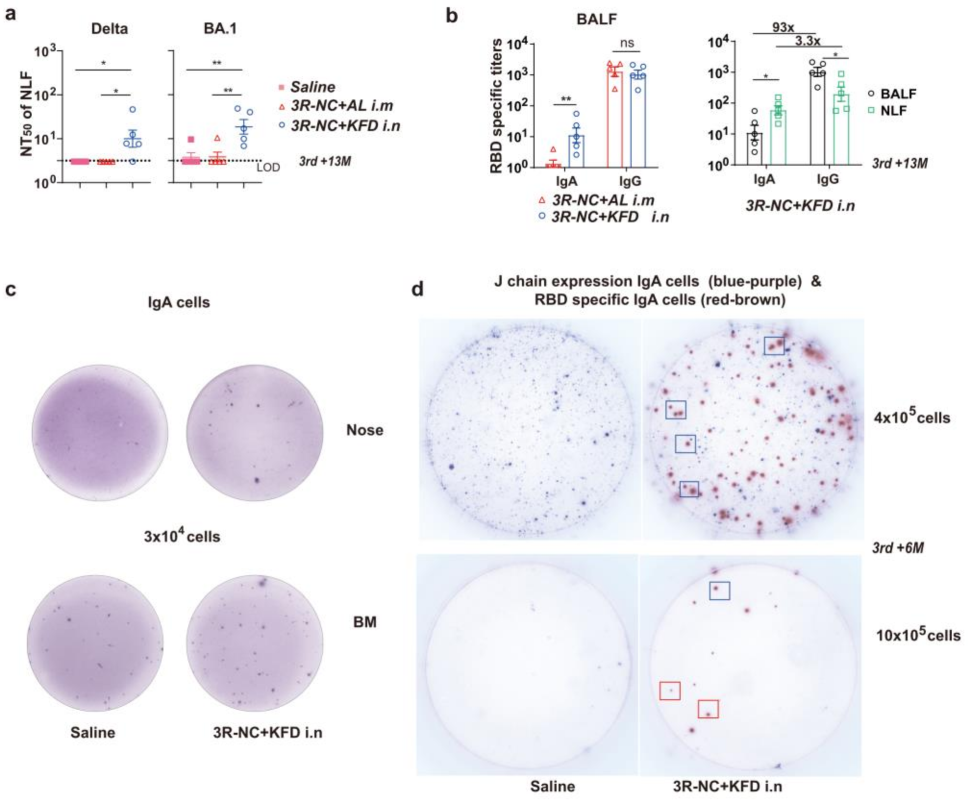

Fig. S5. Antibody responses and B cell responses in *3R-NC+KFDi.n* immunized BALB/c mice. Related with figure 2. (**a**) Neutralizing antibody in NLF against pseudo-typed SARS-CoV-2 variants at 13 months post the 3rd immunization. (**b**) RBD-specific IgG and IgA in BALF at 13 months post 3rd immunization and the comparation with that in NLF. (**c** and **d**) J chain expression and RBD-specificity of IgA secreting cells in nose tissue and bone marrow at 6 months post 3rd immunization. Briefly, wells of plate were coated by the goat anti-mouse IgA antibody to capture the IgA secretion by cultured cells. After 18 hours of cell culturing, cells were removed. (**c**) AP labeled goat anti-mouse IgA was incubated. Next, the BCIP-NBT were used to staining the total IgA secreting cells (blue-purple). (**d**) The wells were incubated with rabbit anti-J chain polyclonal antibody and biotin labeled RBD. Then AP conjugated goat anti-Rabbit IgG and HPR labeled streptavidin were incubated. Next, the BCIP-NBT were used to staining the J chain expressed IgA secreting cells (blue-purple). At last, the AEC were used to staining the RBD-specific IgA secreting cells (red-brown). The co-localizations were labeled by blue squares (red spots with dark brown- black center). Data are represented as mean ± SEM. Groups were compared using one-way ANOVA in (a) or unpaired two-sided Student t-test in (b). $*P < 0.05$; $**P < 0.01$; ns, nonsignificant.

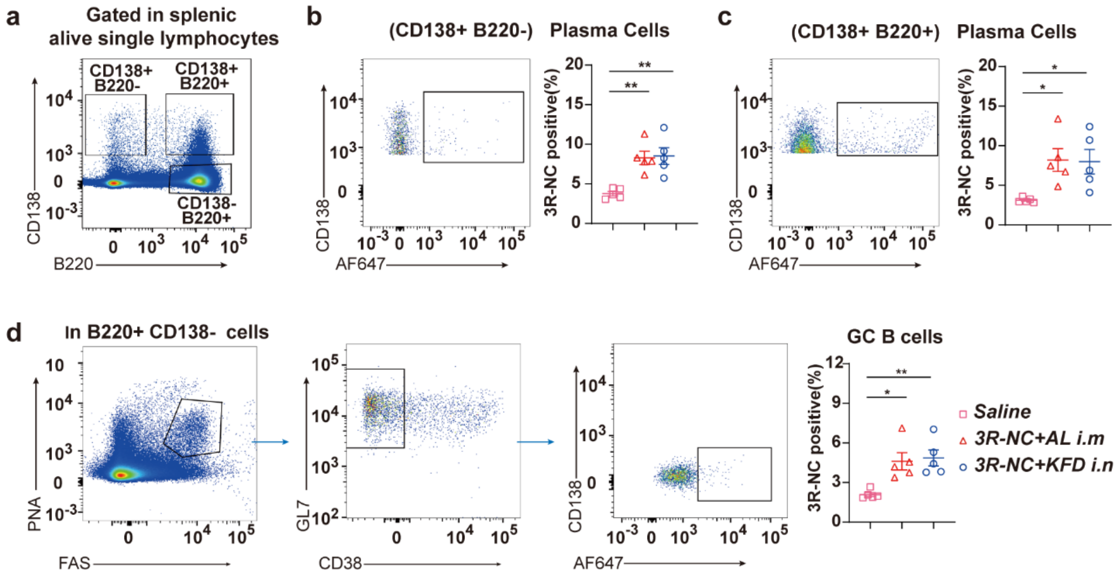

Fig. S6. B cell responses in *3R-NC+KFDi.n* or *3R-NC+ALi.m* immunized BALB/c mice. 3R-NC-AF647 were incubated with splenic cells at day 10 post the 3rd immunization (n=5 mice per group). (**a**) Gating strategy of $B220^{-}CD138^{+}$ plasma cells, $B220^{+}CD138^{+}$ plasma cells and $B220^{+}CD138^{-}$ B cells. (**b-d**) FACS assayed AF647 labeled 3R-NC binding B cells in $B220^{-}CD138^{+}$ plasma cells, $B220^{+}CD138^{+}$ plasma cells and germinal center B cells ($FAS^{+}PNA^{+}GL7^{+}CD38^{-}$ in $B220^{+}CD138^{-}$ cells) respectively. Data are represented as mean ± SEM. Groups were compared using one-way ANOVA. **P* < 0.05; ***P* < 0.01; ns, nonsignificant.

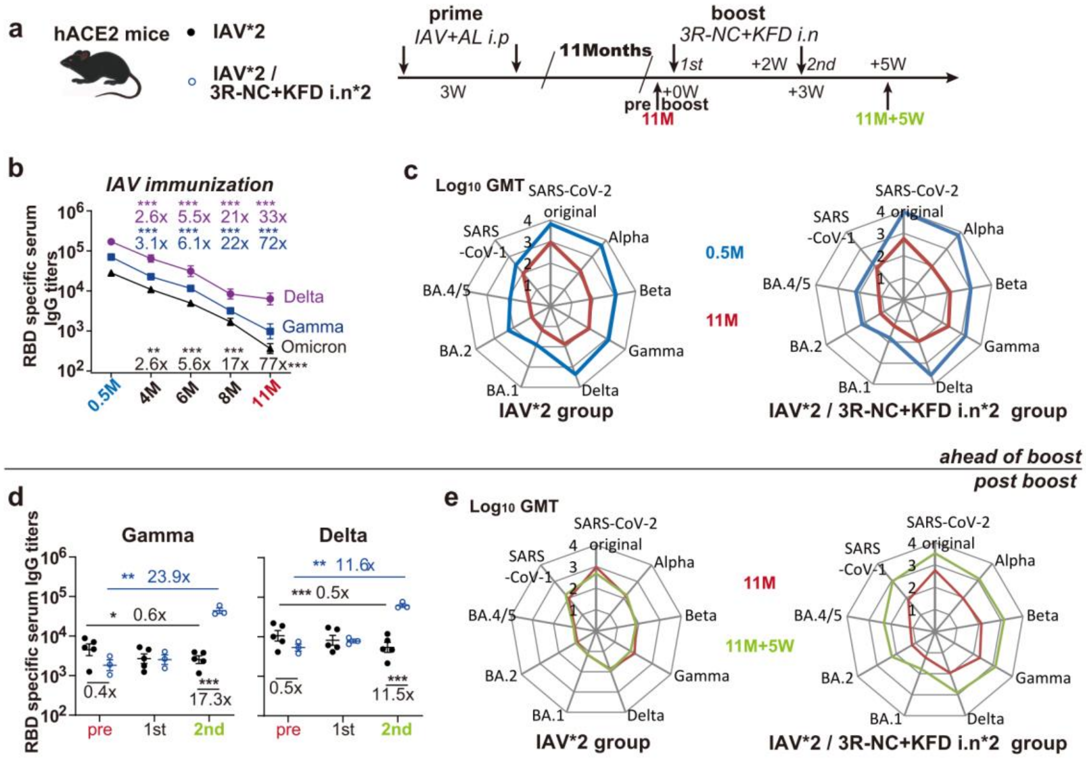

Fig. S7. Boost effect of *3R-NC+KFDi.n* on IAV primed hACE2 mice. (**a**) Diagram scheme of immunization. Two groups of hACE2 mice were primed with two doses of inactivated SARS-CoV-2 virus (IAV). After 11 months, one group of mice (2 female and 1 male) were intranasally boosted with 2 doses of 4μg 3R-NC plus 1μg KFD (*3R-NC+KFDi.n*). The other group of mice (3 female and 2 male) were not boosted. (**b**) RBD-specific serum IgG at indicated time point post the 2nd immunization of IAV. (**c**) Geometric mean of neutralizing antibody titers against pseudo-typed SARS-CoV-2 variants and SARS-CoV-1 at 0.5-month and 11-months post the 2nd immunization. (**d**) RBD-specific serum IgG pre and post the 1st and 2nd dose of *3R-NC+KFDi.n.* (**e**) Geometric mean titers of neutralizing antibody in serum against different pseudo-typed variants of *3R-NC+KFDi.n* boosted and non-boosted mice. Data are represented as mean ± SEM. Groups were compared using one-way ANOVA. **P* < 0.05; ***P* < 0.01; ****P* < 0.001. LOD, limit of detection.

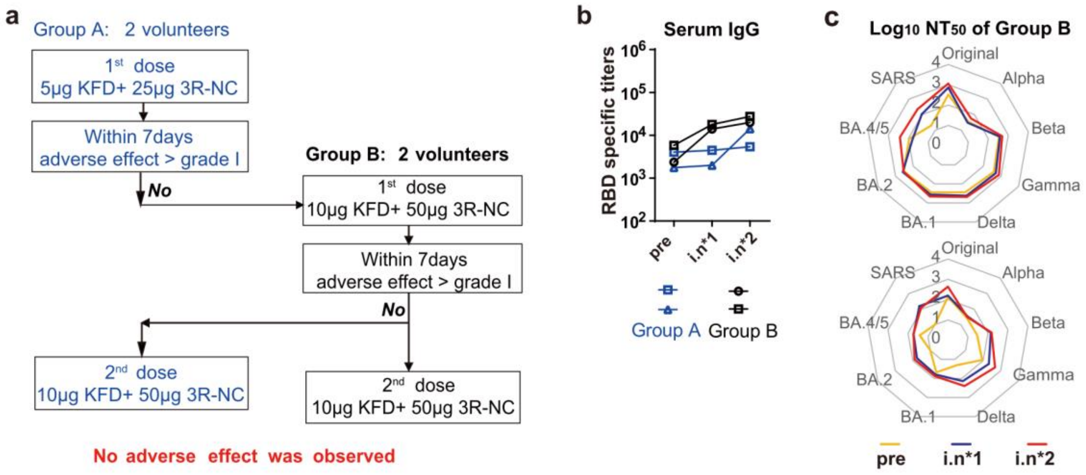

Fig. S8. Identification the minimum effect dose of intranasally sprayed 3R-NC+KFD. (**a**) The schedule to identify the Minimum Effect Dose of 3R-NC+KFD. Four volunteers (25-35 years old), who had received three doses of IAV, were divided into two groups. Two volunteers in Group A received first dose with 25 μg 3R-NC and 5 μg KFD. Within 7 days post inoculation, no adverse effect were observed in these two volunteers. Hence the two volunteers in Group B received their higher dosage (50 μg 3R-NC and 10 μg KFD) as the first dose. In these two volunteers of Group B, no adverse effect were observed within 7 days post inoculation. At last, at 3 weeks post the first dose, volunteers in Group A and Group B received their second dose (50 μg 3R-NC and 10 μg KFD). None adverse effect were observed within 14 days post the 1st and the 2nd doses. (**b**) RBD-specific plasma IgG at time points of pre inoculation, 3 weeks post the 1st and the 2nd doses. (**c**) Neutralizing antibody titers in volunteer's plasma against different pseudo-typed variants pre and post *3R-NC+KFDi.n* boost.

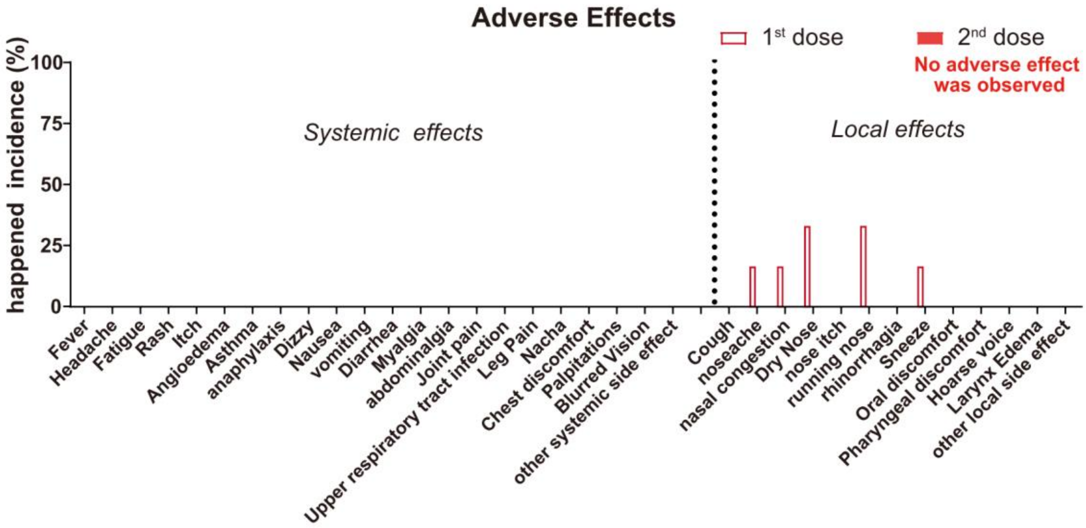

Fig. S9. Safety of *3R-NC+KFDi.n* in the IAV primed volunteers. Related with Fig.5. Adverse effects on the volunteers post the 1st and 2nd doses of 3R-NC plus KFD.

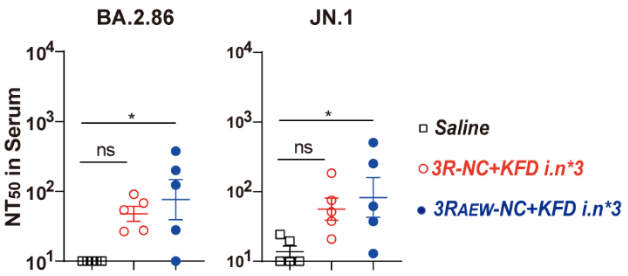

Fig. S10. Neutralizing antibody induced by *3R-NC+KFDi.n* or *3R$_{AEW}$-NC+KFDi.n* in BALB/c mice. Related with Fig.6. Neutralization titers against pseudo-typed SARS-CoV-2 variants BA.2.86 and JN.1, in serum post the 3rd immunization. Data are represented as mean ± SEM. Groups were compared using one-way ANOVA. $*P < 0.05$.

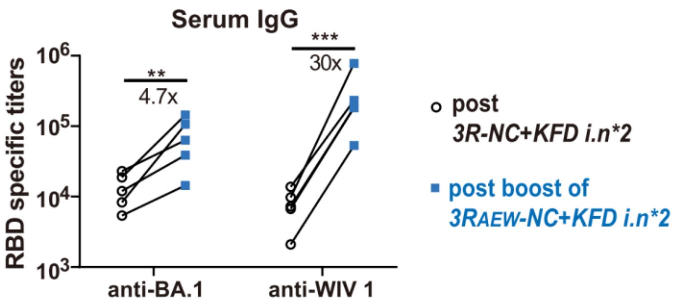

Fig. S11. Boost effects of *$3R_{AEW}$-NC+KFDi.n* on the *3R-NC+KFDi.n* primed BALB/c mice. Related with Fig.6. RBD-specific IgG titers in serum of the mice post the 2 doses of *3R-NC+KFDi.n* prime and post 2 doses of *$3R_{AEW}$-NC+KFDi.n* boost. Data are represented as mean ± SEM. Groups were compared using paired *t*-test. $^{**}P < 0.01$; $^{***}P < 0.001$.

Table S1: Basic Information of the volunteers and side effect post inoculation

Years old	Gender	IAV doses	Basic disease	KFD (μg) + 3R-NC (μg)	Side effect post 1 dose	Side effect post 2 dose
28	Male	3	No	20+80	Dry nose	No
24	Female	2	No	20+80	Nose ache, Nasal congestion	No
23	Male	3	rhinitis	40+160	Running nose	No
29	Male	3	No	40+160	Running nose, Sneeze	No
25	Male	3	No	40+160	Dry nose	No
25	Male	3	No	40+160	No	No
